# Impact of Gene Dosage on Gene Expression, Biological Processes and Survival in Cervical Cancer: A Genome-Wide Follow-Up Study

**DOI:** 10.1371/journal.pone.0097842

**Published:** 2014-05-30

**Authors:** Ingrid Medina-Martinez, Valeria Barrón, Edgar Roman-Bassaure, Eligia Juárez-Torres, Mariano Guardado-Estrada, Ana María Espinosa, Miriam Bermudez, Fernando Fernández, Carlos Venegas-Vega, Lorena Orozco, Edgar Zenteno, Susana Kofman, Jaime Berumen

**Affiliations:** 1 Unidad de Medicina Genómica, Facultad de Medicina, Universidad Nacional Autónoma de México/Hospital General de México, México, D.F. México; 2 Servicio de Oncología, Hospital General de México, México, D.F. México; 3 Servicio de Genética, Hospital General de México/Facultad de Medicina, Universidad Nacional Autónoma de México, México, D.F. México; 4 Laboratorio de Genómica de Enfermedades Complejas, Instituto Nacional de Medicina Genómica. México, D.F. México; 5 Departamento de Bioquímica, Facultad de Medicina, Universidad Nacional Autónoma de México, México, D.F. México; 6 Departamento de Medicina Experimental, Facultad de Medicina, Universidad Nacional Autónoma de México, México, D.F. México; Albert Einstein College of Medicine, United States of America

## Abstract

We investigated the role of tumor copy number (CN)–altered genome (CN-AG) in the carcinogenesis of cervical cancer (CC), especially its effect on gene expression, biological processes, and patient survival. Fifty-nine human papillomavirus 16 (HPV16)-positive CCs were investigated with microarrays–31 for mapping CN-AG and 55 for global gene expression, with 27 CCs in common. Five-year survival was investigated in 55 patients. Deletions and amplifications >2.5 Mb were defined as CN alterations. The %CN-AG varied from 0 to 32.2% (mean = 8.1±8.9). Tumors were classified as low (mean = 0.5±0.6, n = 11), medium (mean = 5.4±2.4, n = 10), or high (mean = 19.2±6.6, n = 10) CN. The highest %CN-AG was found in 3q, which contributed an average of 55% of all CN alterations. Genome-wide, only 5.3% of CN-altered genes were deregulated directly by gene dosage. In contrast, the rate in fully duplicated 3q was twice as high. Amplification of 3q explained 23.2% of deregulated genes in whole tumors (r^2^ = 0.232, p = 0.006; analysis of variance), including genes located in 3q and other chromosomes. A total of 862 genes were deregulated exclusively in high-CN tumors, but only 22.9% were CN altered. This suggests that the remaining genes are not deregulated directly by gene dosage, but by mechanisms induced *in trans* by CN-altered genes. Anaphase-promoting complex/cyclosome (APC/C)-dependent proteasome proteolysis, glycolysis, and apoptosis were upregulated, whereas cell adhesion and angiogenesis were downregulated exclusively in high-CN tumors. The high %CN-AG and upregulated gene expression profile of APC/C-dependent proteasome proteolysis were associated with poor patient survival (p<0.05, log-rank test). Along with glycolysis, they were linearly associated with FIGO stage (r>0.38, p<0.01, Spearman test). Therefore, inhibition of APC/C-dependent proteasome proteolysis and glycolysis could be useful for CC treatment. However, whether they are indispensable for tumor growth remains to be demonstrated.

## Introduction

Cervical cancer (CC) is the second most common cancer in women worldwide, affecting 500,000 individuals each year; it is the leading cause of cancer death among women in developing countries [1]. The viral oncoproteins E6 and E7 of the high-risk human papillomavirus (HPV) play an important role in carcinogenesis. They inhibit various cellular targets, including the tumor-suppressor proteins p53 and pRb, disrupt key cellular processes, such as apoptosis and cell cycle control, and lead to genomic instability and neoplastic development [2]. Despite the damage caused by these oncoviral proteins, CC is a rare complication of the viral infection; most infections are transient and do not evolve into neoplastic lesions. On average, 12–15 years can pass before a persistent HPV infection leads to CC via the premalignant stages of cervical intraepithelial neoplastic lesions [3]. These findings suggest that HPV infection alone does not cause the disease and that other factors, such as abnormal host genes, are associated with the development of invasive cancer.

Genomic imbalances can contribute to deregulated expression of oncogenes and tumor suppressor genes in cancer cells, and the accumulation of such altered genes has been correlated with tumor progression [4]. Several copy number (CN)-altered regions (CNAs) have been identified in CC through tumor genome analysis using methods such as comparative genomic hybridization, fluorescence in situ hybridization, and microarrays (MAs). Gains of 3q [5]–[11] and 5p [5], [12]–[15] are the most frequent chromosomal alterations in CCs, and they have also been described in other solid tumors [16]–[18]. The smallest consensus region of 3q amplification in CC maps to chromosomal cytobands 3q26–27 [6]–[10], [19], suggesting that genes such as *TERC* [20], [21], *PIK3CA* [22], and *ECT2* [23], which are considered candidate oncogenes for CC, may be involved in cervical carcinogenesis. The extent to which these recurrent chromosomal alterations are relevant for tumor development is still largely unknown. On the other hand, the full amplification of 5p is well documented in tumor samples and cell lines [12], [23]–[25]. Some genes amplified in this region and proposed to be involved in CC, such as *SKP2*, *TERT*, *TRIO*, *RNASEN*, and *PRKAA1*, are overexpressed in tumor samples [15] and cell lines [12], [24].

The contribution of CN alterations to cervical carcinogenesis is unresolved owing to a genome-wide lack of correlation between CNAs and gene expression [23], even in completely amplified chromosomal arms such as 3q or 5p [23]–[24], [26]–[27]. In a previous study, we investigated whether CNAs in the cell lines CaLo, CaSki, HeLa, and SiHa are associated with changes in gene expression [23]. Genome-wide, only a small percentage of genes located in CNAs (15.6%) or minimal recurrent regions (18.8%) were deregulated. However, these percentages were, at most, 4% higher than that in the group of genes without CN alterations (14.8%). These data suggest that only approximately 4% of CN-altered genes overall are deregulated directly by gene dosage in CC-derived cell lines. Even in genomic segments confirmed to be entirely amplified, such as 3q and 5p, the percentage of deregulated genes was not always increased. In the case of 5p, the percentage of deregulated genes increased up to 33% in the 4 cell lines. Although 3q was almost entirely amplified in CaLo (93.5%) and HeLa (87.2%), the proportion of deregulated genes increased only approximately 2-fold in HeLa (23.4%) but not in CaLo (12.7%) compared with that in CaSki (13.9%) and SiHa (9.4%), which showed only partial amplification of 3q [23]. Interestingly, not all deregulated genes from duplicated 5p and 3q were overexpressed. Instead, approximately 20% of genes deregulated in 5p and more than 50% of genes deregulated in 3q were downregulated. Therefore, factors other than gene dosage, such as epigenetic mechanisms, may influence gene expression within entirely amplified genome segments. There are no studies that explored the global correlation between the CN alterations and gene expression in CC.

In this study, we explored 59 HPV16-positive CCs with microarrays–31 for mapping CN-AG and 55 for global gene expression, with 27 CCs in common. We investigated genome-wide, on a gene-by-gene level, the proportion of CN-altered genes that are deregulated and the extent of the total altered transcriptome in CC that is deregulated directly or indirectly by gene dosage. We also investigated the biological processes in cervical carcinogenesis that are linked with genes deregulated by gene dosage and the influence of gene dosage on overall patient survival.

## Results

### Overall Tumor Genome Analysis: Identification of Chromosomes, Regions, and Genes Altered in CN

**Figure 1:**
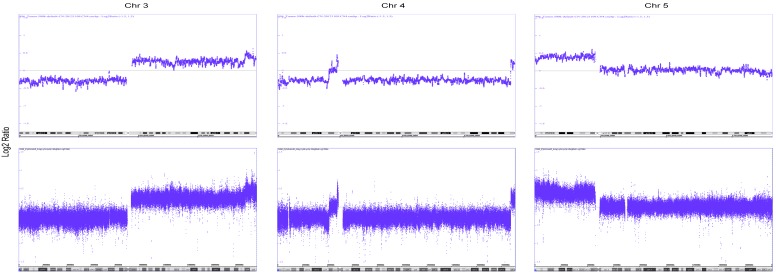
Validation of the GeneChip Human Mapping 500(500 K) microarray with the high-density CytoScan HD microarray. Intensity signals of single nucleotide polymorphisms (SNPs) or non-polymorphic probes are expressed as log_2_ ratios from chromosomes 3, 4, and 5 of the tumor R496 explored using the 500 K microarray (upper panel) and HD 2.7 microarray (lower panel). In both panels, the y-axis depicts a log_2_ ratio scale from –1.5 to 1.5, and the x-axis shows the ideogram of the explored chromosomes with genome positions. The horizontal line crossing the point y = 0 corresponds to 2 copies. The average density of explored positions is more than 5 times higher in the HD 2.7 microarray than that in the 500 K microarray (see materials and methods).

**Figure 2:**
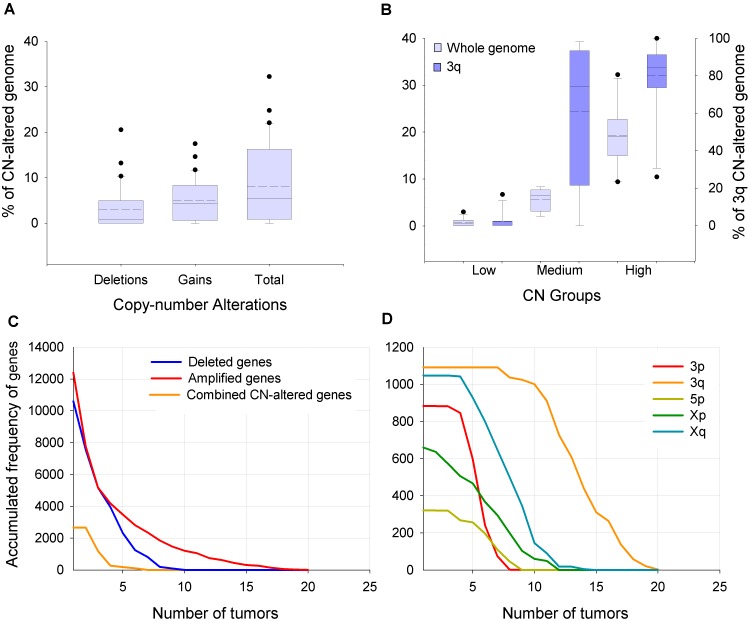
Amount of copy number (CN)-altered genome and frequency of recurrent CN-altered genes. Panel A shows box plots with the distribution of tumors (n = 31) according to the percentage of total, deleted, or amplified CN-altered genome (CN-AG). Panel B shows the distribution of tumors, grouped as low (n = 11), medium (n = 10), and high (n = 10) according to the percentage of global and 3q CN-AG. The horizontal lines inside the boxes represent the median (solid) and average (dotted), and the whiskers represent the minimum and maximum values within the 1.56 interquartile range from the end of the box. Values outside this range are represented by black circles. The decline in the accumulated frequency of recurrent CN-altered genes, as increase the number of tumors that shared the same altered gene, is shown for the whole genome (Panel C) or for chromosomes with significant high %CN-AG (Panel D). Combined genes are those that were deleted in some and amplified in other tumors.

**Figure 3:**
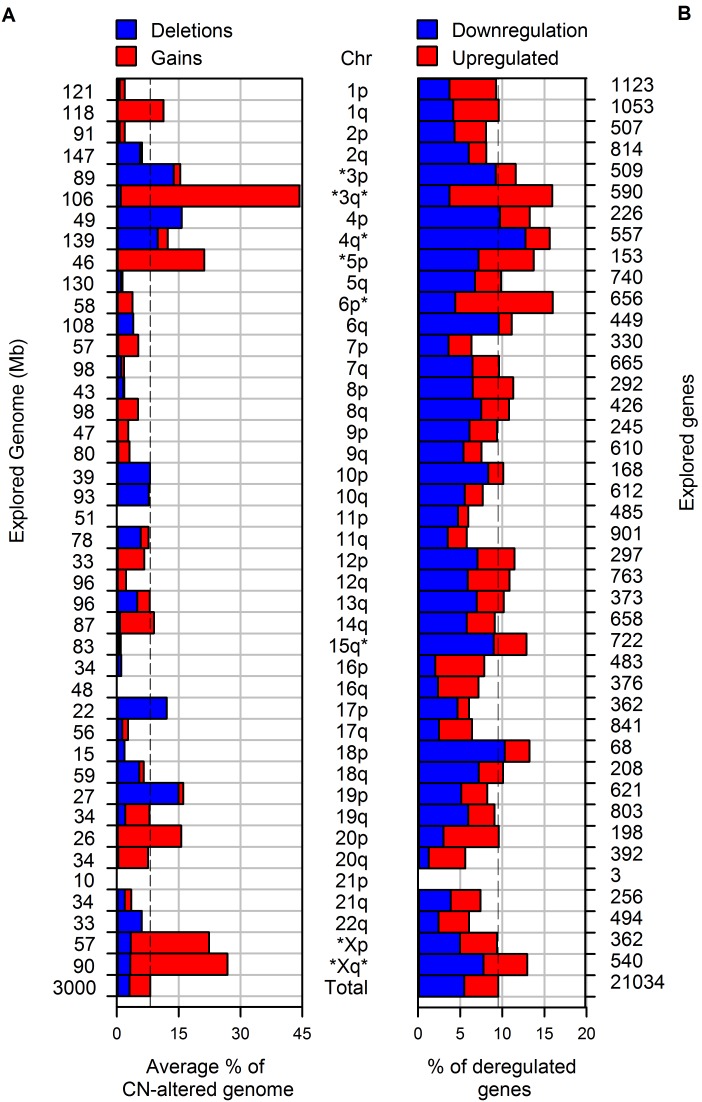
Comparison between CN and gene expression by chromosome arm. The left side shows the explored genome (Mb) in each chromosome arm that was explored with the 500 K microarray in 31 tumors. The right side shows the number of genes on each arm explored with the Human Gene 1.0 ST microarray in 27 of the tumors in which CN was examined. Each bar represents the percentage of CN-AG (left) or deregulated genes (right) of the chromosomal arm indicated in the middle. Red bars indicate gains or overexpression and blue bars represent losses or subexpression. The dotted line represents the average percentage of the global CN-AG (8.1%; left) or the percentage of deregulated genes (9.5%; right) in the tumor genome. The arms marked with asterisks had an average proportion of CN-AG or percentage of deregulated genes superior and statistically significant compared to the numbers found in the complete tumor genome (p<0.05, chi-square).

A total of 673 CNAs larger than 2.5 Mb were identified based on the analysis with the GeneChip Human Mapping 500 K (500 K) microarray: 446 amplifications and 227 deletions. These regions were validated with a second high-density microarray (CytoScan HD2.7) in 15 of the 31 tumors examined with the 500 K microarray. The average coincidence between the 2 arrays was 79.3%, but it increased linearly from 70% in the CNAs of 2.5–3 Mb to 93.4% in CNAs greater than 10 Mb (r = 0.93, p<0.001, Spearman correlation; Figure S1). In fact, when the complete chromosomal arms were compared, the correlation between the 2 microarrays was close to 100% (Figure 1). On average, tumors had 22±19 CNAs (range 0–65). From the size of the haploid genome (3,000 Mb), we calculated the percentage of CN-altered genome (CN-AG) for every tumor. The percentages varied widely among the tumors from 0% to 32.2% (mean = 8.1±8.9%) and followed a nonparametric distribution (Figure 2A, Table 1). Tumors were divided into 3 groups according to %CN-AG: low (mean = 0.5±0.6, n = 11), medium (mean = 5.4±2.4, n = 10), and high (mean = 19.2±6.6, n = 10; Figure 2B, gray boxes). Only 5 chromosomal arms had an average %CN-AG higher than that of the genome average and reached statistical significance (p<0.05, chi-square; marked with an asterisk in Figure 3A). Four of these arms showed mainly gained genome (3q, 5p, Xp, Xq), and only 3p showed a mainly deleted genome. The highest percentage was found in 3q (44.3%), followed well below by Xq (26.8%), Xp (22.4%), and 5p (21.2%; see Figure 3A). Only CN-alterations in chromosomes 3q, Xp and 5p showed a linear correlation with the global alterations in the genome (r = 0.88, p<0.0001, analysis of variance). These 3 chromosomes may explain 76% of the variation in CN (adjusted r2 = 0.76), with 3q ranking at the top and accounting for 55% of all CN alterations in the tumor genome [multiple linear regression (MLR)].

**Table 1:** Influence of copy number alterations in gene deregulation in cervical carcinomas.

			Frequency of deregulated genesa													
			Gene CN status													
	CN-alterations		CN1			CN3			CN1/CN3			CN2			All	
Tumor	% Genome	Genesb	n	EX+	%	n	EX+	%	n	EX+	%	n	EX+	%	EX+	%
																
R035	7.7	3640	301	70	23.3	1799	423	23.5	2100	493	23.5	18936	2178	11.5	2671	12.7
R062	8.3	2737														
R072	5.3	1672	540	44	8.1	467	75	16.1	1007	119	11.8	20027	2284	11.4	2403	11.4
R075	1.2	368	0	0	0	225	25	11.1	225	25	11.1	20809	2183	10.5	2208	10.5
R111	7.7	2976														
R116	3	1033														
R154	7	2554	779	48	6.2	783	113	14.4	1562	161	10.3	19475	1924	9.9	2085	9.9
R183	6.5	2336	897	81	9	522	137	26.2	1419	218	15.4	19615	2577	13.1	2795	13.3
R189	18.6	5778	1558	163	10.5	1646	219	13.3	3204	382	11.9	17833	1875	10.5	2257	10.7
R194*	0	0	0	0	0	0	0	0	0	0	0	21034	2681	12.7	2681	12.7
R198	24.8	8826	2044	163	8	3078	614	19.9	5122	777	15.2	15915	1618	10.2	2395	11.4
R208	0.2	125	0	0	0	63	9	14.3	63	9	14.3	20971	1771	8.4	1780	8.5
R221	0.7	183	62	4	6.5	43	4	9.3	105	8	7.6	20929	1460	7	1468	7
R240	1.5	631	253	13	5.1	127	9	7.1	380	22	5.8	20655	1276	6.2	1298	6.2
R244	0	0	0	0	0	0	0	0	0	0	0	21034	908	4.3	908	4.3
R248	0	0														
R260	3.5	1295	208	14	6.7	560	75	13.4	768	89	11.6	20270	1615	8	1704	8.1
R282	11	4337	1438	144	10	1116	194	17.4	2554	338	13.2	18484	1931	10.4	2269	10.8
R298	19.6	6762	1235	112	9.1	2675	459	17.2	3910	571	14.6	17125	1483	8.7	2054	9.8
R322	9.4	2559	264	20	7.6	1065	91	8.5	1329	111	8.4	19708	1441	7.3	1552	7.4
R365	32.2	10666	3618	551	15.2	2471	381	15.4	6089	932	15.3	14950	1597	10.7	2529	12
R369	0.9	96	0	0	0	52	9	17.3	52	9	17.3	20983	1666	7.9	1675	8
R380*	2.7	862	29	1	3.4	437	63	14.4	466	64	13.7	20568	2062	10	2126	10.1
R386	16.2	5349	723	56	7.7	2248	225	10	2971	281	9.5	18066	1040	5.8	1321	6.3
R392	2	465	51	6	11.8	182	23	12.6	233	29	12.4	20802	1310	6.3	1339	6.4
R444*	0	0	0	0	0	0	0	0	0	0	0	21034	1646	7.8	1646	7.8
R476	0.2	197	145	7	4.8	0	0	0	145	7	4.8	20889	1854	8.9	1861	8.8
R478	1.2	1205	900	27	3	6	1	16.7	906	28	3.1	20129	1041	5.2	1069	5.1
R483	17.1	6030	2407	265	11	1137	138	12.1	3544	403	11.4	17495	1555	8.9	1958	9.3
R494	22.1	6846	2456	521	21.2	1371	199	14.5	3827	720	18.8	17209	2146	12.5	2866	13.6
R496	20.9	5856	1569	445	28.4	1626	264	16.2	3195	709	22.2	17841	2648	14.8	3357	16
Average	8.1	2754	795	102	12.8	878	139	15.8	1673	241	14.4	19362	1769	9.1	2010	9.6

a 21,034 genes were explored in all but four tumors (R062, R111, R116, R248) for changes in expression with HG 1.0 ST microarray. On average 1,673 of them were CN-altered (CN1/CN3) and 19,362 did not have copy number alterations (CN2). CN1 means genes with one copy deleted, CN2 means genes without CN alterations (2 copies), and CN3 means amplified genes with three or four copies.

b Copy number altered genes according to data obtained with 500 K microarray.

n = number of genes identified in the analysis of copy number with 500 K microarray that were also explored for gene expression with the HG 1.0 ST microarray.

EX+ = Genes that were up- or down- regulated in tumors compared with the control sample.

Samples R075 and R189 are ACC and R298 is ASCC, the rest are SCC.

Samples labeled with an asterisk were excluded for the survival analysis.

To identify genes with CN alterations, we aligned CNAs with human genes according to position in the genome. The number of altered genes ranged from 0 to 10,666 (mean = 2,754 genes; see Table 1) among the tumors and correlated positively with %CN-AG (r = 0.99, p<0.0001, Pearson’s correlation). Most CN-altered genes were not shared among the tumors. The accumulated frequency of recurrent CN-altered genes drastically decreased when the number of tumors that shared the same altered gene increased (Figure 2C). In fact, no gene was altered in all 31 tumors explored, and only 3 genes (*RPL21P39*, *NLGN1*, *GM2AP1*) were altered in 20 tumors. The 3q genes were the most recurrent −up to 10 tumors displayed alteration of nearly 100% of the explored genes−, and the 3q curve was shifted to the right 6 tumors from the nearest curve of the other chromosomes (see Figure 2D). A total of 264 genes had recurrent alterations, all from 3q, in 16 (51.6%) or more tumors.

**Figure 4:**
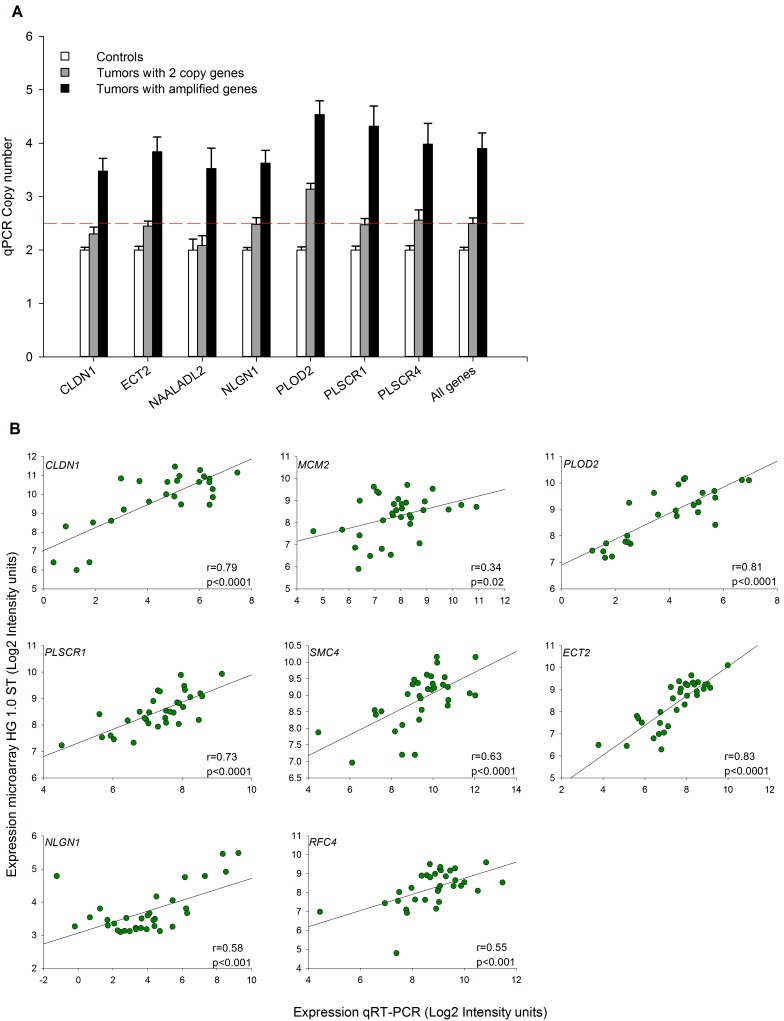
Validation of genes amplified and deregulated located in 3q by qPCR and qRT-PCR. The top panel shows the average copy number of 7 genes (*CLDN1*, *ECT2*, *NAALADL2*, *NLGN1*, *PLOD2*, *PLSCR1*, and *PLSCR4*) located in 3q explored with qPCR in controls (lymphocytes) and tumors with 2 or 3–4 copies identified with the 500 K microarray. Whiskers of each bar represent the standard error of the mean. The dotted red line shows the value for 2.5 copies calculated with qPCR (see material and methods). The bottom panel shows the correlation of gene expression of 8 genes (*MCM2*, *PLOD2*, *PLSCR1*, *SMC4*, *ECT2*, *NLGN1*, *RFC4*, and *CLDN1*) located in 3q explored in 27 tumors and 6 controls with both the HG 1.0 ST microarray and qRT-PCR techniques. Log_2_ values of the normalized intensity signals obtained with the microarray (robust multichip average values) and qRT-PCR were plotted. Trend line (black line), correlation coefficient (r), and p value were calculated with Pearson’s correlation test.

We selected 7 of the most recurrent genes located on 3q (*CLDN1*, *ECT2*, *NAALADL2*, *NLGN1*, *PLOD2*, *PLSCR1*, and *PLSCR4*) to be validated with a real-time quantitative polymerase chain reaction (qPCR) technique in all 31 tumors and 17 controls. All genes displayed a significant positive correlation (r>0.6, p<0.0001, Pearson’s correlation) between the mean intensity (log2 ratio) of SNPs identified with the microarray in the CNAs, in which the genes were located in each tumor, and the number of copies calculated with qPCR (data not shown). When the number of copies calculated with qPCR was compared between the groups, the average CN of the 7 explored genes was significantly higher in tumors that had alterations than in those without alterations in these 3q genes (p<0.05, t test; Figure 4A).

### Analysis of Global Gene Expression

**Figure 5:**
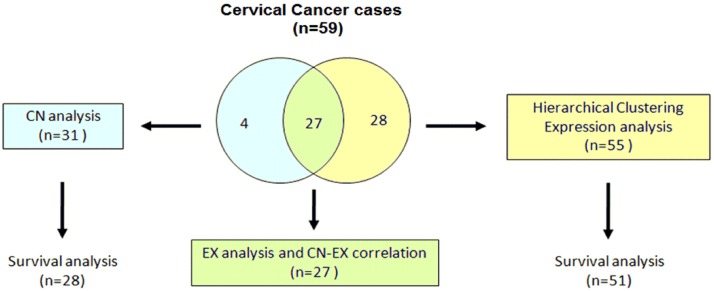
Analysis workflow of 59 cervical cancer cases. Figure shows the analysis workflow of the 59 CC cases explored in this study. All CC samples were HPV16 positive and were investigated with microarrays–31 for mapping CN-AG and 55 for global gene expression, with 27 CCs in common. These 27 CCs were used for the analyses of global gene expression and the correlation between the CN-AG and gene expression. For the hierarchical clustering analysis, the expression profiles of the 55 CC samples were included. Five-year survival was investigated in 55 patients, 51 explored for gene expression and 28 for CN alterations, with 24 CCs in common. See material and methods section for details of the procedures.

The amount of messenger RNA (mRNA) transcribed from 21,034 genes was explored using the microarray HG 1.0ST in 27 of the 31 tumors examined with the 500 K microarray and in 17 normal cervical epithelium controls (Figure 5, Table 1 and Table 2). We identified 2,006 altered genes (9.5%), 57.6% downregulated and 42.4% upregulated (Table S1). When the 2 adenocarcinomas (ACC; R075 and R189 in Table 1) and the adenosquamous cell carcinoma (ASCC; R298 in Table 1) were excluded from the analyses, we found a similar number of genes and concordance of 95% with the list of altered genes. Therefore, to maintain sample size we include all 27 CCs.

**Table 2:** Patients followed up on average for 63 months for survival evaluation.

Sample	Histology^a^	Tumor Stage	Age (years)	Treatment^b^	Follow up (months)	Status^c^	Analysis performed^d^
R035	SCC	IB2	48	TELE+CHEMO+HT	73	Alive	CN/EX
R062	SCC	IB2	42	TELE+CHEMO+HT	62	Alive	CN
R072	SCC	IB1	61	HT+TELE+BRACHY	86	Alive	CN/EX
R075	ACC	IB1	73	TELE+HT	58	Alive	CN/EX
R081	ACC	IB1	41	HT	62	Alive	EX
R102	SCC	IIB	66	TELE+CHEMO+HT	84	Alive	EX
R111	SCC	IB1	44	TELE+HT	78	Alive	CN
R116	SCC	IB1	30	HT	26	Death	CN
R121	SCC	IB1	35	HT	61	Alive	EX
R124	SCC	IB1	42	HT	62	Alive	EX
R154	SCC	IIIB	42	TELE+CHEMO	26	Death	CN/EX
R156	SCC	IIIB	70	TELE+BRACHY+CHEMO_i_+HT	6	Death	EX
R157	SCC	IVB	69	PALIATIVE CARE	2	Death	EX
R158	SCC	IVB	37	PALIATIVE CARE	12	Death	EX
R183	SCC	IB1	64	TELE+BRACHY	70	Alive	CN/EX
R189	ACC	IB1	47	HT	60	Alive	CN/EX
R194	SCC	IIA	64	UNTREATED	19	Death	CN/EX
R198	SCC	IVA	50	PALIATIVE CARE	10	Death	CN/EX
R208	SCC	IB1	46	TELE+BRACHY	23	Death	CN/EX
R221	SCC	IB2	41	TELE+BRACHY+CHEMO	33	Death*	CN/EX
R230	SCC	IB1	62	TELE+BRACHY	71	Alive	EX
R240	SCC	IIIB	31	TELE+BRACHY+CHEMO	11	Death	CN/EX
R244	SCC	IIB	52	TELE+HT	83	Alive	CN/EX
R248	SCC	IB1	36	TELE+BRACHY+HT	24	Death	CN
R260	SCC	IB2	24	TELE+CHEMO	66	Alive	CN/EX
R265	SCC	IB1	46	TELE+BRACHY	67	Alive	EX
R268	SCC	IIB	34	TELE+BRACHY+CHEMO	58	Alive	EX
R282	SCC	IIIB	41	TELE+BRACHY+CHEMO	70	Alive	CN/EX
R298	ASCC	IIIB	50	TELE+CHEMO+HT	48	Death	CN/EX
R311	SCC	IIIB	72	UNTREATED	1	Death	EX
R315	SCC	IIIB	41	TELE+BRACHY+CHEMO	10	Death	EX
R322	SCC	IIIB	74	TELE+BRACHY	60	Alive	CN/EX
R324	SCC	IB2	28	TELE+BRACHY+CHEMO	14	Death	EX
R330	SCC	IB1	72	TELE+BRACHY	54	Alive	EX
R336	SCC	IB2	36	TELE+BRACHY+CHEMO	64	Alive	EX
R339	SCC	IB2	31	TELE+BRACHY+CHEMO	13	Death	EX
R365	SCC	IIIB	38	TELE+CHEMO	6	Death	CN/EX
R368	SCC	IIIB	36	TELE+BRACHY+CHEMO_i_	70	Alive	EX
R369	SCC	IB1	50	HT	65	Alive	CN/EX
R378	SCC	IB2	42	TELE+BRACHY+CHEMO	56	Alive	EX
R380	SCC	IIIB	64	UNTREATED	12	Death	CN/EX
R386	SCC	IB1	73	TELE+BRACHY	70	Alive	CN/EX
R390	SCC	IB1	51	TELE+BRACHY+CHEMO_i_	64	Alive	EX
R392	SCC	IIIB	69	TELE+BRACHY	61	Alive	CN/EX
R409	SCC	IB2	68	TELE+BRACHY+CHEMO	42	Alive	EX
R411	SCC	IB1	34	HT+TELE+BRACHY	60	Alive	EX
R412	SCC	IB2	33	TELE+BRACHY+CHEMO	63	Alive	EX
R426	SCC	IIB	67	TELE+BRACHY+CHEMO	23	Death	EX
R444	SCC	IIB	71	UNTREATED	26	Death	CN/EX
R455	SCC	IIA	67	TELE+BRACHY	60	Alive	EX
R457	SCC	IIIB	60	TELE+BRACHY	15	Death	EX
R476	SCC	IB1	41	HT	68	Alive	CN/EX
R478	SCC	IB1	46	TELE+BRACHY	60	Alive	CN/EX
R483	SCC	IVB	51	PALIATIVE CARE	11	Death	CN/EX
R486	SCC	IB1	39	TELE+HT	33	Alive	EX
R489	SCC	IIIB	43	TELE+BRACHY	37	Alive	EX
R494	SCC	IIIB	55	TELE+CHEMO+BRACHY_i_	6	Death	CN/EX
R495	SCC	IIB	68	TELE+BRACHY	62	Alive	EX
R496	SCC	IIIB	52	TELE+BRACHY+CHEMO	63	Alive	CN/EX

a ACC, Adenocarcinoma. SCC, Squamous Cell Carcinoma. ASCC, Adenosquamous Cell Carcinoma.

bHT, Radical Hysterectomy. Tele, teletherapy. Brachy, brachytherapy. Chemo, chemotherapy with Cisplatin. i. Means incomplete treatment.

c Status alive at the last follow up record and death was caused by primary tumor of cervical cancer, except the case labeled with an asterisk. The cause of death was unknown.

d CN indicate the samples analyzed for CN (500 K array), CN/EX indicate the samples analyzed for CN (500 K array) and gene expression (HG 1.0 ST array), and EX indicate the samples analyzed for gene expression (HG 1.0 ST).

The frequency of deregulated genes was calculated by chromosome. Of the 42 explored arms, only 5 (3q, 4q, 6p, 15q, and Xq) showed a higher and statistically significant percentage of deregulated genes compared with the overall percentage (9.5%; marked with an asterisk in Figure 3B). The chromosomes that showed the highest percentage were 6p (16.0%, p<0.001) and 3q (15.9%, p<0.001), followed by 4q (15.6%, p<0.001), Xq (13.0%, p<0.02), and 15q (12.9%, p<0.04); chi-square test for all comparisons. Upregulated genes predominated in 3q and 6p, whereas downregulated genes were most prevalent in 4q, 15q, and Xq (see Figure 3B).

We evaluated the expression of 28 genes selected for validation with a qRT-PCR technique in 27 CC samples and 6 controls (Table S2). We found a significant positive correlation (average r = 0.74, p<0.05; Pearson’s correlation) between the logarithmic values (log2) of the data obtained with qRT-PCR and the microarray in 27 of the 28 genes explored (96.4%). Figure 4B shows the correlation of the intensity values (log2) obtained with the 2 methods for 8 genes located on 3q. These data indicated that the expression values calculated with the microarray were fairly reliable because up to 96.4% of validated genes had a significant correlation.

To identify the biological processes associated with the 2,006 differently expressed genes, we used the Database for Annotation, Visualization, and Integrated Discovery tool (DAVID; http://david.abcc.ncifcrf.gov/). Compared with the human genome database, the 5 most enriched clusters with the lowest p values at medium stringency were cell cycle including mitosis, DNA metabolic processes including DNA repair, chromosome segregation, cytoskeleton organization, and developmental processes (Table 3). Interestingly, at the highest stringency, where more tightly associated genes in each group are expected, the clusters including mitosis were ranked first, third, and fifth (biological processes in italics in Table 3).

**Table 3:** DAVID functional annotation cluster analysis in the 2006 genes differentially expressed in cervical cancer.

Cluster*	Regulation	Biological process	# Genes	p-value	FC
1	+	cell cycle	207	1.20E-38	2.5
	+	*mitosis (1)*	84	6.10E-27	3.6
2	+	DNA metabolic process	122	8.40E-19	2.3
	+	DNA repair	68	1.50E-10	2.3
3	+	chromosome segregation	36	3.60E-14	4.2
4	+/−	cytoskeleton organization	83	1.20E-07	1.8
5	−	developmental process	428	7.50E-10	1.3
	−	cell differentiation	209	1.80E-03	1.2
6	+	regulation of cell cycle	76	8.90E-11	2.2
	+	cell cycle checkpoint	29	1.10E-07	3
7	−	response to endogenous stimulus	81	1.90E-08	1.9
8	+	meiotic cell cycle	31	7.10E-08	2.9
9	+	DNA packaging	44	4.60E-14	3.6
	+	chromosome organization	97	6.30E-10	1.9
10	−	angiogenesis	34	2.40E-05	2.2
12	+	interphase	28	1.00E-05	2.5
22	−	*cell migration (2)*	49	3.40E-04	1.7
23	+	*mitotic cell cycle spindle assembly checkpoint (3)*	6	3.50E-03	5.2
	+	*negative regulation of mitotic metaphase/anaphase transition (3)*	6	3.50E-03	5.2
24	+	DNA strand elongation during DNA replication	5	5.70E-04	9.5
25	+	DNA replication initiation	10	9.10E-06	5.9
	+	*DNA geometric change (4)*	6	3.50E-02	3.2
28	+	double-strand break repair	19	4.70E-05	2.9
29	+	regulation of DNA metabolic process	27	1.10E-04	2.2
	+	*DNA replication checkpoint (6)*	4	3.00E-02	5.4
33	−	regulation of cell adhesion	28	1.00E-03	1.9
36	+	*regulation of ubiquitin-protein ligase activity during mitotic cell cycle (5)*	16	6.50E-03	2.1
	+	anaphase-promoting complex-dependent proteasomal ubiquitin-dependent protein catabolic process	14	1.70E-02	2
	+	negative regulation of ubiquitin-protein ligase activity	14	2.10E-02	2
39	−	epidermal cell differentiation	15	1.70E-02	2
41	+	nucleobase, nucleoside, nucleotide and nucleic acid metabolic process	201	1.10E-05	1.3
52	NC	*regulation of chemotaxis (7)*	8	3.90E-02	2.4

*The cluster number was obtained when the analysis was run with the whole gene set, including up- and down-regulated genes.

FC = Fold change is the ratio of the proportion of genes in the tested list versus the Human Gene Reference database.

NC =  No clustered in up (+) and down (−) regulated genes analyzed separately.

The clusters in italics were enriched when the functional annotation cluster analysis was run at the highest stringency, and the number inside the parenthesis indicated the order the cluster occupied in the list.

**Figure 6:**
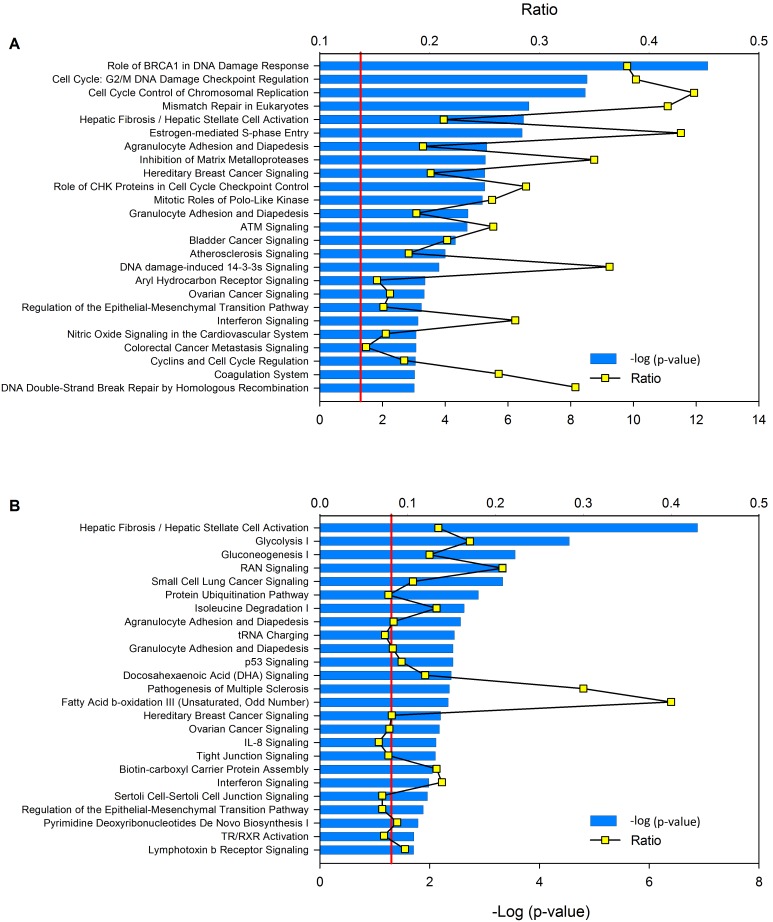
Canonical pathways involving deregulated genes. Panel A shows the top 25 canonical pathways identified in the set of 2,006 deregulated genes in the whole set of tumors. Panel B shows the top 25 canonical pathways identified in the set of 862 genes deregulated exclusively in high-CN tumors. The canonical pathways were identified with the Ingenuity Pathway Analysis software. The -log (p value) and ratio were calculated by comparing the number of genes belonging to the pathway present in the data sets with the human genome database. The p value was calculated using the chi-square or Fisher’s exact test, as appropriate, and values of -log (p value) higher than 1.3 (red line) correspond to a value of p<0.05. The canonical pathway of hypoxia inducible factor (HIF1α) signaling was statistically significant in the deregulated genes in the whole set (43rd place) and in the high-CN set of tumors (26th place).

The total group of deregulated genes was also analyzed with Ingenuity Pathway Analysis (IPA) software, and the overall results were very similar to those obtained with the DAVID tool (data not shown). Of the 89 canonical pathways that IPA identified as altered (p<0.05, Fisher exact test), those involved in the cell cycle and DNA repair ranked at the top of the list (Figure 6A).

### Correlation between Changes in CN and Gene Expression

A simple analysis of Figure 3 suggests that no clear correlation exists between gene expression and CNAs. For example, of the 5 chromosomes with the highest percentage of CN-AG, only 2 (3q, Xq) showed a significant increase in the percentage of deregulated genes. Furthermore, the other 3 chromosomes showing a significant increase in deregulated genes (4q, 6p, 15q) lacked a high percentage of CN-AG, even 6p and 15q had a percentage well below the overall average. Although overexpressed genes predominate in most of the chromosomes with a high percentage of gains, these chromosomes also have a high proportion of downregulated genes, and in some, such as Xq, the proportion of downregulated genes predominates (Figure 3). On the other hand, chromosomes that displayed predominantly deletions (3p, 4p) also had a percentage of upregulated genes, although the percentage of downregulated genes predominated.

To understand more thoroughly the relationship between CN alteration and gene expression, we analyzed these variables gene by gene in each tumor. The expression status (downregulated, upregulated, or without change) for each explored gene (n = 21,034) was identified in each tumor using cutoff values (see materials and methods). The CN status of each of these genes was also identified in each tumor. The number of genes with and without changes in CN and gene expression is shown for each tumor in Table 1. The average number of CN-altered genes was 1,673, and the average number of 2 copy genes was 19,362 (see Table 1). On average, 2,010 (9.6%) genes were deregulated in tumors, of which 241 were CN altered and 1,769 had 2 copies. These data mean that, on average, only 14.4% (241/1,673) of the CN-altered genes and 9.1% (1,769/19,362) of 2 copy genes were deregulated. The difference between the 2 subgroups was only 5.3% (p<0.00001, chi-square) and corresponds to the average fraction of genes with CN alterations that may be deregulated directly by gene dosage. Notably, only 69% of the amplified and deregulated genes were overexpressed; the rest were downregulated. Furthermore, only 82% of the deleted and deregulated genes were downregulated; the rest were overexpressed (data not shown). These results agree with observations at the chromosome level.

**Figure 7:**
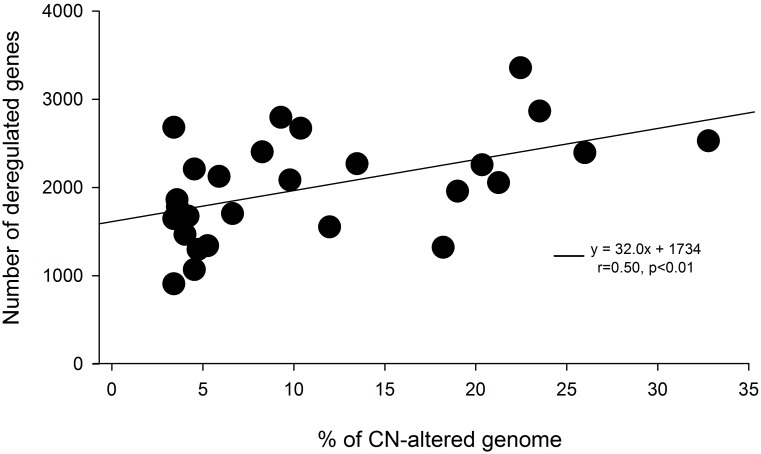
Trend of deregulated genes with increased %CN-AG. The trend of the number of deregulated genes with increased %CN-AG in the whole set of 21,034 explored genes are shown. The line represents the linear correlation trend for the data set. Also shown are the equation of the line, the correlation coefficient, and p value calculated with the Pearson’s correlation test.

To investigate whether a linear trend exists between gene expression and the amount of CN alteration (CN-AG), we analyzed the correlation between the 2 variables, including the individual data for all tumors studied. As expected, the total number of deregulated genes increased with %CN-AG (calculated from Table 1, r = 0.5, p = 0.007, Pearson’s correlation; Figure 7). According to the equation in Figure 7 (y = 32x+1,734), in a tumor with 0% CN-AG (x = 0), the number of deregulated genes is approximately 1,734 and would be deregulated by mechanisms other than gene dosage. By contrast, the tumor with the highest %CN-AG (R365 = 32.2%, see Table 1) will have an additional 1,034 deregulated genes (total = 2,768). This number is very close to the observed number of deregulated genes (see Table 1) and included the fraction of deregulated genes that may be deregulated directly by gene dosage. In this extreme case, the number corresponds to 37.4% of all deregulated genes (1,034/2,768). In the whole set of tumors, only 12% (241/2,010) of deregulated genes on average were CN altered (calculated from Table 1). Only CN-alterations in 3q showed a clear linear regression with global gene expression (r = 0.51, p = 0.006, analysis of variance), and it explained 23.2% (adjusted r^2^ = 0.232, MLR) of all changes in gene expression.

### Identification of Deregulated Genes and Biological Processes Associated with Changes in Gene Dosage

#### a) Deregulated genes in low- and high-CN tumors

To identify the differentially expressed genes in tumors with low and high percentages of CN alterations (see Figure 2B, gray boxes), we compared each group of tumors with the control group (n = 17) using the SAM method. Of the 21,034 explored genes, 1,757 (8.4%) were deregulated in tumors with high CN, but only 1,104 genes (5.2%) were deregulated in tumors with low CN. Interestingly, 895 (81.1%) of the deregulated genes in the low-CN group also were deregulated in the high-CN group. The difference in the number of deregulated genes between the high-CN group and the common genes (n = 862) corresponded to the fraction of genes deregulated by gene dosage in the high-CN group (49.1%).

Remarkably, only 9.2% of the genes deregulated exclusively in high-CN tumors (79 of 862) were amplified (n = 76) or deleted (n = 3) in 6 or more tumors. The remaining genes were CN altered in 4–5 (n = 118), 1–3 (n = 370), or no (n = 295) tumors (Table S3). Based on these calculations, we conservatively assumed that in this group of genes exclusively associated with high-CN tumors (n = 862), gene dosage has a direct influence on gene expression in, at most, 22.9% of these genes (CN altered in ≥4 tumors). The remaining genes (CN altered in ≤3 tumors, n = 665) would not be deregulated directly by gene dosage but by other mechanisms, perhaps influenced *in trans* by some CN-altered genes.

#### b) Identification of biological processes associated with tumors with high and low CN

**Figure 8:**
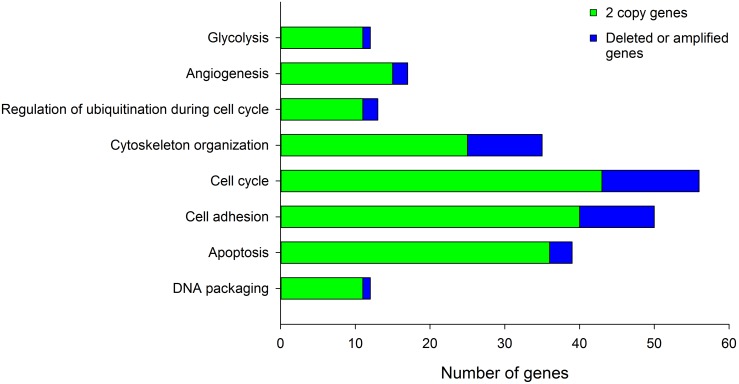
Identification of CN-altered genes in biological processes enriched in high-CN tumors. Shown are the numbers of 2-copy or CN-altered genes in ≤3 tumors (green bars) and CN-altered genes in ≥4 tumors (blue bars) among the biological processes enriched in the subset of genes deregulated exclusively in high-CN tumors.

The DAVID tool was used to identify the biological processes enriched in the subset of common genes (n = 895) and those deregulated exclusively in high-CN (n = 862) and low-CN (n = 209) tumors. The clusters of biological processes found in the set of common genes were very similar to those enriched in the whole set of tumors (Table 3 vs. Table S4). However, some important biological processes enriched in the whole set of tumors, such as angiogenesis, regulation of cell adhesion, and the anaphase-promoting complex/cyclosome (APC/C)-dependent proteasomal ubiquitin-dependent protein catabolic process (see Table 3) were not enriched in the subset of genes shared by both groups. Interestingly, these 3 processes were enriched in the high-CN tumors; moreover, they ranked first among the biological processes (Table 4). Other clusters enriched exclusively in the high-CN group but not identified in the subset of common genes included glycolysis, which ranked second, apoptosis, and mRNA transport (see Table 4). These data suggest that these processes are closely linked to gene dosage. The processes of cytoskeleton organization, cell cycle, and DNA packaging, although not exclusive, were also enriched in the group of genes associated with high-CN tumors, suggesting that some genes involved in these processes can be deregulated by gene dosage. All these biological processes, except cell adhesion and angiogenesis, were associated with overexpressed genes. Interestingly, at the highest stringency, the processes of APC/C-dependent proteasomal ubiquitin-dependent protein catabolic process ranked first, followed by glycolysis and DNA packaging (biological processes in italics in Table 4). Notoriously, most of the genes involved in the biological processes associated with high-CN tumors were not CN altered (Figure 8). On the other hand, only 2 clusters were enriched in the subset of genes exclusively deregulated in low-CN tumors: cell differentiation and the processing and presentation of peptide antigen via major histocompatibility complex class I (data not shown). This suggests that most of the genes involved in these 2 processes are deregulated through mechanisms other than gene dosage.

**Table 4:** DAVID functional annotation cluster analysis of genes expressed exclusively in High-CN tumors.

Cluster	Regulation	Biological process	# Genes	p-value	FC
1	−	tube development	21	3.00E-03	2.1
2	+	*glycolysis (2)*	12	7.10E-06	5.5
	+	*carbohydrate catabolic process (2)*	12	1.20E-02	2.4
3	−	blood vessel development	24	1.00E-03	2.1
	−	angiogenesis	17	1.30E-03	2.5
4	+	*anaphase-promoting complex-dependent proteasomal ubiquitin-dependent protein catabolic process (1)*	13	3.60E-05	4.3
	+	*regulation of ubiquitin-protein ligase activity during mitotic cell cycle (1)*	13	8.90E-05	4
5	+/−	cytoskeleton organization	35	2.00E-03	1.7
6	+	cell cycle	56	9.70E-04	1.6
	+	mitotic cell cycle	27	2.20E-02	1.6
7	−	cell adhesion	50	2.40E-03	1.5
8	+	cellular component organization	142	4.90E-03	1.2
9	+	cell death	50	4.10E-03	1.5
	+	apoptosis	39	3.10E-02	1.4
10	+	*nucleosome assembly (4)*	11	5.20E-03	2.8
	+	DNA packaging	12	1.90E-02	2.2
19	+	*mRNA transport (9)*	10	1.90E-02	2.5
	+	nucleobase, nucleoside, nucleotide and nucleic acid transport	11	3.60E-02	2.1
20	+	*carboxylic acid metabolic process (8)*	37	2.60E-02	1.4
24	−	*mesenchymal cell differentiation (5)*	8	8.60E-03	3.4

See the legends in Table 3.

Of the set of clusters in italics, which were obtained with the analyzed at highest stringency, the clusters number 3 (female gonad development), 6 (hair cycle process) and 7 (amino acid activation) were not identified in the table because those biological processes were not represented in the analysis at medium stringency and have not previously been associated with cancer.

**Figure 9:**
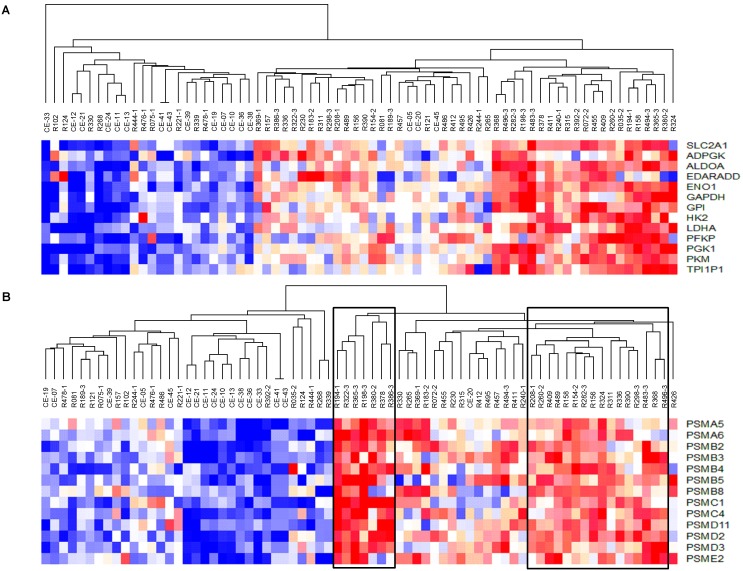
Segregation of tumors and control samples according to gene expression profile. Unsupervised hierarchical cluster analysis of 55 CCs and 17 healthy cervical epithelium samples using the expression values of the genes deregulated from glycolysis (panel A) and the anaphase-promoting complex/cyclosome (APC/C)-dependent proteasomal protein catabolic process (panel B) obtained with the HG 1.0 ST microarray. Each row represents a gene and each column represents a sample. Samples name beginning with an “R” are CCs and with a “C” are controls; CCs ending in 1, 2 or 3 belong to low-, medium- or high-CN groups, respectively, whereas those ending with no number were not explored for CN. The length and the subdivision of the branches represent the relationships among the samples based on the intensity of gene expression. The cluster is color-coded using red for upregulation, blue for downregulation, and white for unchanged expression. In panel B, the sub-branches enclosed in squares were considered together in a group with a strong upregulation profile and the remaining upregulated samples were placed in another group with a weak upregulation profile.

To investigate whether the gene expression profiles of the biological processes associated with high-CN tumors allow the segregation of tumors either by CN or by biological processes themselves, we performed an unsupervised hierarchical clustering to classify 55 tumors explored with the HG 1.0 ST microarray (Table 2), including the 27 tumors explored for CN analysis, and 17 healthy cervical controls. Only the expression profiles of glycolysis and APC-dependent proteasomal protein catabolic process clearly segregated the tumors in groups with specific expression profiles (Figure 9). In the analysis of glycolysis, the dendrogram showed 3 main branches: 1 with a strong upregulated profile, another with a combination of weak up- and downregulated signals, and a third with a strong profile of downregulation. These results show the heterogeneity in the gene signature of this biological process in the whole set of samples. Most tumors (81.8%) clustered evenly into the first 2 branches, and a minority clustered in the third branch (18.2%). By contrast, all but 3 controls were clustered in the third branch. Notably, high-CN tumors were grouped in the strongly (60%) and combined (40%) upregulated profiles, whereas the low-CN tumors grouped mainly in the downregulated (50%) and combined (30%) profiles (p = 0.028, Fisher exact test; Figure 9A).

In the analysis of the APC-dependent proteasomal protein catabolic process, the dendrogram showed two main branches: 1 with a downregulated profile, and 1 with an upregulated profile. However, the branch with the upregulated profile showed several sub-branches, 3 of which contained samples with strong signals (Figure 9B, enclosed in squares) and the remainder composed of samples with weak signals (see Figure 9B). All but 1 sample in the upregulated branch were tumors, and only 1 control was segregated in this branch. By contrast, most controls (94.1%) and 30.9% of tumors were segregated into the downregulated branch. Interestingly, 90% of the high-CN tumors were grouped in the weakly (10%) or strongly (80%) upregulated profiles, whereas the low-CN tumors were mainly grouped in the downregulated profile (60%; p = 0.023, Fisher exact test; see Figure 9B).

### Analysis of Genes and Biological Processes Related to 3q

**Figure 10:**
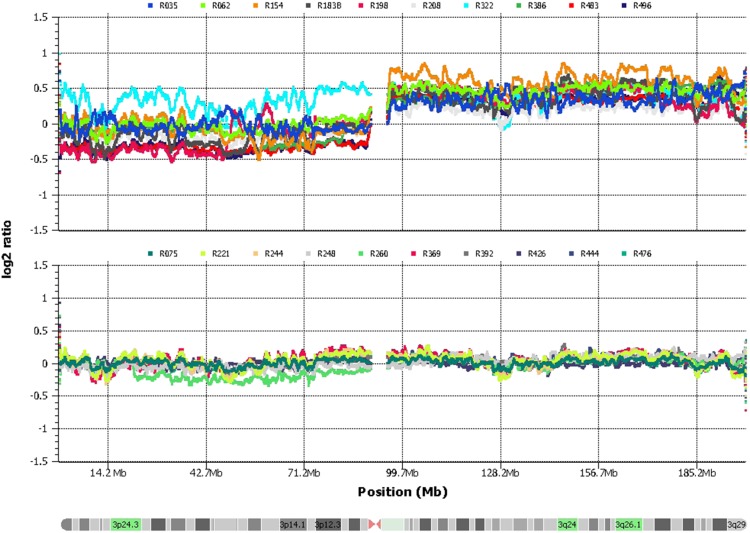
Genome amplification in chromosome 3q. Shown is the CN log_2_ ratio of SNPs investigated in chromosome 3 with the 500 K SNP microarray in tumors with duplicated 3q (n = 10, upper panel) and normal 3q (n = 10, lower panel). The intensity (log_2_ ratio) was plotted using the SVS software (Golden Helix). Genome positions are indicated in millions of base pairs (Mb).

The expression of 3q genes was compared between high- and low-CN tumors. The median percentage of amplified genome on 3q was 84% in tumors with high CN and 0% in tumors with low CN. Figure 10 shows the intensity (log2 ratio) of studied chromosome 3 SNPs in tumors with and without 3q amplification. Although most of 3q was duplicated in the first group, only 101 of the 590 explored genes (17.1%) were deregulated (82 upregulated and 19 downregulated). In tumors with no 3q amplification, only 41 genes were deregulated (6.9%; 33 upregulated and 8 downregulated; Table S1). Therefore, duplication of 3q increased 2.5 times the number of deregulated genes. All but 4 deregulated genes (*BCHE*, *CD80*, *MIR15B*, and *TNFSF10*) in low-CN tumors were also deregulated in high-CN tumors (Table S1). Interestingly, the group of common genes were which had the higher FC in high-CN tumors; *MCM2* had the highest increase in gene expression, followed by *ECT2*, *RFC4*, *POLQ*, and *PLOD2*, all of them with a FC of >4. Notably, the expression of these genes was higher in tumors with 3q amplification (Table S1), indicating that the amplification of 3q further enhanced the upregulation of these common genes. Genes implicated previously in CC, such as *TERC* and *PIK3CA*, were notably not deregulated in the high-CN tumors, even when they were amplified.

According to MLR analysis, 3q amplification must also be involved in the deregulation of genes located on other chromosomes. Therefore, linear correlation analyses were performed between the %CN-AG of 3q and the intensity (robust multichip average) of gene expression of the 862 genes deregulated exclusively in high-CN tumors, including all 27 tumors explored for CN alterations and global gene expression (Table 1). Only 192 genes showed a significant correlation (p<0.05, Pearson’s correlation; Table S1). Therefore, the total involvement of 3q in the high-CN tumors includes these 192 genes and the 101 deregulated genes located in 3q.

The biological processes in which this set of genes are involved were investigated with the DAVID tool. Processes previously associated with high-CN tumors (for example, cell adhesion, DNA packaging, the APC/C-dependent proteasomal ubiquitin-dependent protein catabolic process, and glycolysis) were enriched (Table 5). The biological process of positive regulation of the I-kappaB kinase/nuclear factor (NF)-kappaB cascade, which was not previously identified, was enriched and ranked tenth. However, in the analysis at highest stringency, this process was enriched in the first place, followed by cellular ion homeostasis and the APC/C-dependent proteasomal ubiquitin-dependent protein catabolic process (see Table 5 and Table S5).

**Table 5:** DAVID functional analysis of genes correlated and non-correlated with 3q amplification in high-CN tumors.

Cluster	Regulation	Biological process	# Genes	p-value	FC
A. Deregulated genes associated with 3q amplification: 101 located in 3q and 192 located in other chromosomes.					
1	NC	cell adhesion	21	5.70E-03	1.9
2	+	chromatin organization	14	5.80E-03	2.4
3	+	cellular protein metabolic process	53	3.50E-03	1.5
4	+	DNA packaging	6	3.40E-02	3.3
	+	*nucleosome assembly (6)*	5	4.00E-02	3.9
5	NC	regulation of DNA metabolic process	6	3.10E-02	3.4
	NC	regulation of DNA repair	3	4.40E-02	8.8
7	NC	*sphingomyelin metabolic process (8)*	3	1.20E-02	18
8	+	*anaphase-promoting complex-dependent proteasomal ubiquitin-dependent protein catabolic process (3)*	5	1.80E-02	5
	+	*regulation of ubiquitin-protein ligase activity during mitotic cell cycle (3)*	5	2.40E-02	4.6
10	NC	*positive regulation of I-kappaB kinase/NF-kappaB cascade (1)*	6	1.70E-02	4
11	+	*cellular ion homeostasis (2)*	12	3.00E-02	2.1
12	NC	*positive regulation of establishment of protein localization to plasma membrane (4)*	3	2.30E-03	39
14	+	*glycolysis (7)*	4	3.50E-02	5.5
22	+	*positive regulation of macromolecule metabolic process (5)*	21	4.10E-02	1.6
B. Deregulated genes non-associated with 3q amplification (n = 606)					
1	−	blood vessel development	19	1.20E-03	2.4
	−	angiogenesis	13	3.50E-03	2.7
	+	*cell migration (5)*	19	4.40E-03	2.1
2	−	response to chemical stimulus	62	1.90E-03	1.5
3	+	cell projection assembly	9	5.80E-03	3.3
4	+/−	cytoskeleton organization	28	1.20E-03	2
5	+	cell death	38	4.40E-03	1.6
	+	apoptosis	29	4.00E-02	1.5
6	+	chordate embryonic development	22	3.10E-03	2
7	+	*mRNA transport (8)*	10	7.70E-03	3.1
8	−	*mesenchymal cell differentiation (3)*	7	6.20E-03	4.2
9	+	*glycolysis (4)*	8	7.90E-04	5.2
	+	*glucose catabolic process (4)*	8	2.80E-03	4.2
10	−	cellular component biogenesis	53	6.30E-04	1.6
11	+	*tRNA aminoacylation for protein translation (6)*	7	3.70E-03	4.6
14	+	*anaphase-promoting complex-dependent proteasomal ubiquitin-dependent protein catabolic process (7)*	8	5.30E-03	3.7
	+	*regulation of ubiquitin-protein ligase activity during mitotic cell cycle (7)*	8	8.50E-03	3.4
NC	−	*ovulation cycle process (1)*	9	9.00E-04	4.4
NC	−	*female gonad development (2)*	8	4.80E-03	3.8

See the legends in Table 3.

Interestingly, the remaining processes, such as angiogenesis, apoptosis, and mRNA transport, that were associated with high-CN tumors but not clustered with 3q-associated genes, were enriched in the complementary subset of genes deregulated exclusively in high-CN tumors but not correlated with 3q amplification (n = 606; see Table 5 and Table S5). However, the regulation of ubiquitin protein ligase activity during the mitotic cell cycle, the APC/C-dependent proteasomal ubiquitin-dependent protein catabolic process, and glycolysis were also notably enriched in this set of genes, suggesting that these processes are linked to both 3q- and non-3q-associated genes.

### Overall Survival Rate of Patients According to the Percentage of CN Alterations and Gene Expression Profiles of Biological Processes Linked to High-CN Tumors

**Figure 11:**
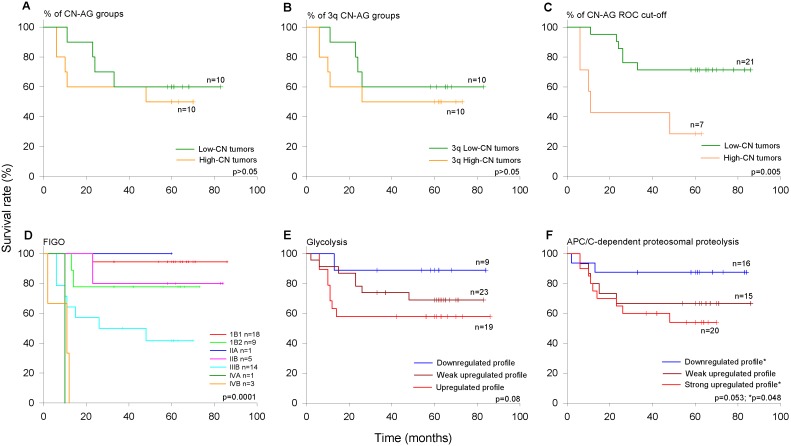
Survival analysis of women with CC according to International Federation of Gynecology and Obstetrics (FIGO) staging, CN-AG, and gene expression profiles. The Kaplan-Meier curves for FIGO staging, the whole and 3q %CN-AG, and gene expression profiles of genes involved in glycolysis and APC/C-dependent proteasomal protein catabolic process (see Figure 9) are shown. Patients were followed up an average of 63 months. The p value was calculated by comparing the curves with the log-rank test. Censored patients are labeled with transverse lines.

A survival analysis was performed using the percentage of CN alterations in the whole genome and 3q chromosome and International Federation of Gynecology and Obstetrics (FIGO) staging in patients with CC who were followed up for at least 63 months after their diagnosis and initial treatment. Three of the 31 patients explored for CN alterations, which refused to be treated, were excluded from the follow-up (Table 2 and Figure 5). This subset included FIGO stages IB1 (n = 12), IB2 (n = 4), IIB (n = 1), IIIB (n = 9), IVA (n = 1), and IVB (n = 1). The overall survival rate was 64.3% and for patients who died, the mean time from diagnosis to death was 19 months. The FIGO stages IB1, IB2, IIB, IIIB, and IV were associated with survival rates of 75%, 100%, 100%, 44.4%, and 0%, respectively. These differences were statistically significant (p<0.05, log-rank test; data not shown). When the %CN-AG was categorized into low (n = 10) and high (n = 10), the survival rate was similar between the groups (66.7 vs. 50%; p>0.05, log-rank test; Figure 11A). Even when the whole set of tumors was categorized in 2 subsets according to %CN-AG (n = 14), the comparison between the groups showed no difference in survival rate (data not shown). The results were very similar in a comparison of the extreme groups according to 3q amplification (Figure 11B). However, when tumors were categorized with the cutoff value of %CN-AG calculated with receiver operating characteristics analysis, the difference was statistically significant (p = 0.005, log-rank test; Figure 11C). In fact, 71.4% of patients with the highest %CN-AG (mean = 22.2%; n = 7) died, whereas only 23.8% of the remaining patients (mean CN-AG = 4.5%; n = 21) died.

Because glycolysis and the APC/C-dependent proteasomal protein catabolic process were the biological processes most linked to high-CN tumors, and tumors clearly segregate according to these gene expression profiles, we investigated survival in a greater number of patients assessed for gene expression but not all for CN alterations (Table 2 and Figure 5). Four of the 55 patients explored for gene expression, which refused to be treated, were excluded from the follow-up (Table 2 and Figure 5). The survival rate by FIGO stage was similar to that found in the subset of 28 tumors explored for CN, and the differences were statistically significant (p<0.05, log-rank test; Figure 11D). Interestingly, the survival of patients with upregulation profiles for either glycolysis (Figure 11E) or the APC/C-dependent proteasomal protein catabolic process (Figure 11F) was much lower than that of patients with downregulation profiles. However, only the difference between the strongly upregulated and downregulated profiles of the APC/C-dependent proteasomal protein catabolic process was statistically significant (p<0.05, log-rank test). When FIGO was included as a covariate in the same Cox proportional hazard model, neither the highest %CN-AG nor the APC-dependent proteasomal protein catabolic process remained significant (data not shown). In fact, a positive correlation was found between FIGO stage and %CN-AG (r = 0.54, p = 0.003), glycolysis (r = 0.4, p = 0.002) and APC/C-dependent proteasomal protein catabolic process (r = 0.39, p = 0.004) (Spearman test).

## Discussion

In this study, a low correlation between CN alteration and changes in gene expression was found. The difference in the percentage of deregulated genes in the group of genes with (14.4%) and without (9.1%) CN changes was only 5.3%. This value would be the percentage of CN-altered genes deregulated directly by gene dosage in CCs. This percentage is similar to that in recurrent altered genes in cell lines derived from CC (3.9%) [23]. However, in fully duplicated chromosomes such as 3q, the total rate of deregulated genes increased to 17.8%. As in cell lines [23], the amplified genes in the tumors were not always overexpressed; instead, they were often downregulated, and 1-copy deleted genes were often overexpressed. Furthermore, variations in gene dosage seem to influence not only the expression of genes directly amplified or deleted but also the expression *in trans* of genes without CN alteration that are located far away, even in other chromosomes. According to the MLR, only the amplifications in 3q showed a linear regression with gene expression, and the overall percentage of genes directly or indirectly deregulated by 3q amplification was approximately 23%. However, in the high-CN tumors, up to 50% of genes were deregulated directly or indirectly by changes in gene dosage, mainly from 3q. We also found that CN-altered genes were mainly involved in the anaphase transition–especially APC/C-dependent proteasomal proteolysis–, glycolysis, apoptosis, cell adhesion and angiogenesis. In addition, high percentages of CN alterations and upregulated profiles of APC/C-dependent proteasomal proteolysis and glycolysis were associated with lower overall survival and advanced FIGO stages. As these findings were discovered in a CC sample mainly composed of SCC (90%), we do not know if they are also valid for ACC and ASCC.

No studies have explored the global correlation between CN and gene expression in CC, and only a few previous reports have examined the correlation in certain chromosomes or regions, specifically 5p [15], 20q [28], and various segments of the genome [26], [29]. Reports on these correlations, similar to the present study, found a low correlation between CN and gene expression. Our study is the first to identify biological processes involving CN-altered genes throughout the tumor genome of CC. The SAM method detected a group of deregulated genes without CN alterations that were associated exclusively with high-CN tumors, suggesting that these genes are deregulated *in trans* or by epigenetic mechanisms induced by the products of other CN-altered genes. The amplifications showed a higher correlation with changes in gene expression than deletions, suggesting that amplifications are a more important mechanism for upregulation than are deletions for downregulation in CC.

As in this study, 3q gain has been the most common alteration found in CC [6], [9], [30], [31]. Nevertheless, no studies of CC have correlated CN alteration in 3q with global gene expression. In the high-CN tumors, 3q accounts for up to 34% of all deregulated genes: only one-third of those genes were located in 3q, the rest were located in other chromosomes. Despite our model not being completely clean, the comparison of 3q gene expression between the extreme tumors (with and without 3q duplication) allowed us to identify genes that were deregulated in 3q primarily by gene dosage or by other mechanisms. The facts that a group of 3q genes were deregulated in both high- and low-CN tumors (37%; 37/101), suggest these genes are likely deregulated primarily by mechanisms other than gene dosage; although gene dosage could have a direct additive effect in upregulated genes. Furthermore, the fact that 19% (19/101) of the 3q genes deregulated in high-CN tumors were downregulated, suggests that in those entirely amplified regions, epigenetic mechanisms could be involved in gene repression.

Genes deregulated in both groups of tumors, rather than those deregulated exclusively in high-CN tumors, are which could be the candidate oncogenes for cervical cancer. The group was headed by *MCM2*, *ECT2*, and *RFC4*, which are associated with DNA replication, DNA repair or positive regulation of signal transduction, indicating that these genes are essential for tumor growth. The levels of upregulation of *MCM2*, *ECT2*, and *RFC4* in both groups of tumors are notable (Table S1); the 3 genes have been reported in CC [32]. We recently found that the *MCM2*, *ECT2*, and *RFC4* genes are overexpressed in 4 cell lines (SiHa, CaSki, HeLa, and CaLo), indicating that these markers are correctly predictive of CC [23]. However, because the amplification recurrence of *ECT2* (n = 18) was much higher than for *MCM2* (n = 13) and *RFC4* (n = 10), it is a good candidate for the oncogene for CC in 3q26, that may be upregulated by gene amplification or another mechanism.

As in this paper, cell cycle is the main altered process in CC and is top ranked in all reports that have analyzed biological processes in CC [33]. In addition, the results of this paper suggests that mitosis is the primary cell cycle phase altered in CC. These findings are consistent with the alterations in the cell cycle and mitosis caused by HPV *in vitro* [34]–[36] and are correlated in other studies of CC [34]. The E6 and E7 oncoproteins of high-risk HPVs induce numerous mitotic defects, including multipolar mitoses, chromosomal missegregation, anaphase bridges, and aneuploidy. Although cells with abnormal mitoses are normally targeted for cell death, E6 and E7 act cooperatively to allow these cells to accumulate by relaxing the G2/M checkpoint response and inhibiting apoptotic signaling [36]. In agreement with these data, the canonical pathway of G2/M DNA damage checkpoint regulation ranked at the second position on the list of altered canonical pathways in CC (Figure 6). Furthermore, E6 and E7 induce mechanisms for mitosis checkpoint avoidance. The *E6*/*E7* genes have been shown to induce the overexpression of *CDC20* and *UBCH10*, which activate the APC/C ubiquitin ligase complex [37]. The control of mitotic metaphase/anaphase transition seems to be essential for tumor progression, and in CC, progress through anaphase and exit of mitosis are likely induced by viral proteins.

In this context of experimental evidence, it is not surprising that the APC/C-dependent proteasomal protein catabolic process was the most enriched in high-CN tumors. Most genes involved in this process seem to be overexpressed by an indirect effect of gene dosage. From the 13 genes overexpressed in high-CN tumors, only 2 of them, *PSMD2* (3q) and *PSMC4* (19q), were amplified in 6 and 4 of 10 tumors with high CN, respectively. The remaining genes were altered in 1, 2, or no tumors (data not shown). These data again suggest that overexpression of these genes could be regulated *in trans* by other genes altered in the CN. The amplified genes encode regulatory proteins that contribute to the assembly of the 26S proteasome.

Other genes involved in the regulation of the APC/C, such as *MAD2L1*, *CDK1*, *BUB1B*, *PSMB9*, *CCNB1*, *UBE2C*, and *CDC20*, were also overexpressed in the full set of deregulated genes in high-CN tumors. However, they were also shared with low-CN tumors, suggesting that the main difference between these groups of tumors is the activation of proteasomal components of APC/C-dependent proteolysis, which ensures the destruction of proteins such as mitotic cyclins and securin that is necessary for the mitosis exit and cell cycle progression. In fact, the association of the upregulated APC/C-dependent proteolysis profile with poor survival suggest this process is close linked to tumor progression.

The increase of glycolysis in high-CN tumors was accompanied by the downregulation of angiogenesis, suggesting that tumors operate under hypoxic conditions. Glycolysis provides the tumor cell with ATP in a less efficient manner than that produced by oxidative phosphorylation; however, the rate of ATP production by anaerobic glycolysis can be up to 100 times faster than that of oxidative phosphorylation (Pasteur effect). To a great extent, this metabolic shift results from the altered expression of the genes and proteins that regulate glycolysis and oxidative phosphorylation. These gene expression changes in other tumors have been shown to be induced, at least in part, by the altered activities of key transcription factors, including NF-kappaB and hypoxia inducible factor (*HIF1A*), together with the loss of tumor suppressor p53, which promotes oxidative phosphorylation and inhibits the Warburg effect [38]. Although the level of *TP53* expression in high-CN tumors was unchanged, the degradation of p53 protein by HPV E6 oncoprotein in CC has been well documented [39]; therefore, it could be a mechanism that facilitated decreased oxygen consumption and increased glycolysis [40]. HIF1A is a key protein involved in the activation of glycolytic enzymes under hypoxic tumor conditions. Although this gene was not overexpressed in high-CN tumors under the cutoff parameters with the SAM method, its expression was 1.4-fold higher in these tumors compared with that in control samples, a difference that was statistically significant with the t test (p<0.05). Furthermore, the canonical pathway of HIF1α signaling was enriched in the full set of genes associated with high-CN tumors (see legend in Figure 6B), suggesting that HIF1A protein is active. Despite the supposed activation of HIF1A protein, the angiogenesis process was downregulated in high-CN tumors, which could be explained by the downregulation of pro-angiogenic genes such as *KDR* and *ANG* and the upregulation of angiogenic inhibitors such as *MMP14* and *ANGPT2*. The I-kappaB kinase/NF-kappaB signaling pathway may orchestrate the aerobic-anaerobic switch given that it was enriched and associated with 3q amplification. NF-kappaB is an important regulator of immune and inflammatory responses and plays a crucial role in multiple cellular pathways including cell survival, proliferation, adhesion, and angiogenesis [41]. NF-kappaB is induced by a wide variety of stimuli, including low oxygen tension (hypoxia) [41], and the loss of p53 [38]. Kawauchi et al. [40] have reported that upon loss of p53, NF-kappaB induces expression of the glucose transporter GLUT3 in tumor cells, increasing glucose consumption and lactate production.

Several other glucose transporters and glycolytic enzymes are often overexpressed in malignant tumors [42]. In this study, we found a glucose transporter gene (*SLC2A1*) and 12 genes associated with glycolysis upregulated exclusively in high-CN tumors, including those that encode 3 regulatory glycolytic enzymes –hexokinase (*HK2*), phosphofructokinase (*PFKP*), and the pyruvate kinase (*PKM*)– indicating that glycolysis is accelerated in these tumors. Furthermore, lactate dehydrogenase (*LDHA*) was also overexpressed in high-CN tumors, suggesting that under the hypoxic conditions of these tumors, most pyruvate is converted to lactate. This step allows the restoration of NAD+ and the continuous flux of glycolysis. Moreover, *LDHA* plays an important role in the aerobic-anaerobic switch and may drive anaerobic tumor metabolism [43]. Interestingly, in these tumors, the *SLC9A1* gene, a pH_i_ regulatory gene, was overexpressed. This gene encodes a Na+/H+ antiporter located in the plasma membrane that plays a central role in regulating pH homeostasis by eliminating acids generated by active metabolism [44]. This process may be fundamental for clearing cellular lactate and driving anaerobic metabolism. This gene is involved in cellular ion homeostasis, a process that was also increased and linked to 3q amplification.

Increases in mitosis, glycolysis, and anabolic metabolism suggest tumor growth. However, the unexpected increase in the expression of genes related to apoptosis, such as members of the superfamily of tumor necrosis factor receptors (*TNFRSF10A*, *TNFRSF10D*, *TNFRSF21*, and *TNFRSF12A*), as well as the downregulation of the anti-apoptotic gene *BCL2*, suggest that contradictory forces are playing key roles in the balance of tumor growth in high-CN tumors. Nevertheless, the lower survival of patients whose tumors had the highest percentage of CN alterations compared with that of patients with tumors with lower percentages suggests that the balance of cellular metabolism in high-CN tumors is favorable for tumor growth rather than cell death.

The fact that the %CN-AG and upregulation of APC/C-dependent proteasomal ubiquitin-dependent protein catabolic process are not independent of FIGO stage in the survival study, indicates that they are not better prognostic markers for CC. In spite of this, the co-linearity between FIGO stage and CN, glycolysis and APC/C-dependent proteasomal ubiquitin-dependent protein catabolic process, suggests that the high %CN-AG and upregulation of these biological processes are associated with more aggressive tumors. Therefore, inhibition of these processes could be a potential therapeutic strategy to combat CC. However, whether they are indispensable for tumor growth remains to be demonstrated.

## Materials and Methods

### Ethics Statement

The study protocol was approved by the scientific and ethics committees of the Hospital General de Mexico (approval number DIC/03/311/04/051) and was performed in accordance with the ethical principles described in the 1964 Declaration of Helsinki. Informed written consent was obtained from all participants before their inclusion in the study.

### Subjects, Samples, and Experimental Design

The study subjects included 59 patients with invasive CC diagnosed in the Department of Oncology and 17 women with normal cervical epithelium evaluated in the Department of Obstetrics and Gynecology at the Hospital General de México in Mexico City. The CC samples were a subset selected from a total of 462 patients with CC who were recruited sequentially from November 2003 through July 2007. Owing to the restrictive inclusion criteria (no previous treatment, incident case, born in Mexico with Mexican ancestry for 2 generations), the samples represented approximately 80% of patients newly diagnosed with CC during this period. The selection criteria for the CC subset were based on the availability of a fresh tumor biopsy for RNA extraction with more than 70% tumor cells in the morphological analysis and positivity for HPV16. Among the samples, 55 were squamous cell carcinomas, 3 samples were adenocarcinomas, and 1 was an adenosquamous carcinoma. The average age of patients was 50.5 years (range, 24–74 years). All patients received complete clinical evaluations. The tumors of CC patients were staged according to the last international revised protocol for gynecologic cancer [45].

Two biopsies, conducted under colposcopy examination, were taken from the tumors. One biopsy was divided into 2 equal parts: 1 part was fixed in buffered formol for morphological analysis and the other part, together with a second biopsy, was snap frozen on dry ice and stored at −80°C until analysis. Five milliliters of blood were obtained from all patients, and DNA was extracted from lymphocytes for use as a control for DNA microarrays. Control cervical specimens were obtained from patients undergoing hysterectomy due to myomatosis. They were previously diagnosed with a normal cervix through cytology and colposcopy. Immediately after receiving a cervix fragment from the operating room, we dissected the exocervical epitheliums under a stereoscopic microscope to avoid stromal cells. The tissues were then snap frozen in liquid nitrogen and stored at −80°C until use. For HPV detection and typing, scrapings from the endocervix and ectocervix were collected with a cytobrush from patients and controls, and the cells were suspended in a vial with extraction buffer and stored at −20°C until analysis.

Global genome analysis (500,568 SNPs) was performed in DNAs extracted from 31 fresh tumor biopsies and 25 lymphocyte samples of cancer patients using the 500 K microarray. Alterations in CN were validated in 15 tumor samples with a second high-throughput microarray (HD 2.7 Cytoscan microarray). The amplification of 7 genes was validated with qPCR in all 31 tumor samples. Analysis of global gene expression (21,034 genes) was performed in RNAs extracted from 55 fresh tumor biopsies, including 27 of those explored for CN, and 17 samples of normal cervical epithelium using the HG 1.0 ST microarray. The expression of 28 genes was validated with qRT-PCR in 27 CC and 6 controls. The association of CN alteration and gene expression profiles with survival was investigated through survival analysis of 51 patients with CC who were followed up for an average of 63 months (Figure 5).

### DNA and RNA Isolation

DNA was purified from cervical scrapings, biopsy, and lymphocyte specimens using a PureLink Genomic DNA Kit (Invitrogen, Grand Island, NY, USA) and maintained at −20°C until analysis. Total RNA was isolated from half of the divided biopsy using TRIzol reagent (Invitrogen) according to the manufacturer’s protocol. The quality of the RNA was confirmed with agarose gel electrophoresis, as demonstrated by the presence of intact ribosomal RNA, with the 28s band twice as intense as the 18s band.

### Detection and HPV Typing

HPV detection was performed with PCR using universal primers located in the HPV *L1* gene *MY09*/*MY11*, *GP5*+/*6+*, and *L1C1* as described previously [46]–[48]. The *HBB* gene was used as an internal control to assess the quality of DNA. The HPV types were identified by sequencing as previously described [49]. Only HPV16 positive samples were included in this study.

### GeneChip Mapping 500 K

The 500 K SNP array analyzed 500,568 SNPs with a mean inter-marker distance of 5.8 kb. Array experiments were performed according to the Affymetrix GeneChip Mapping 500 K standard protocols (Affymetrix Inc., Santa Clara, CA, USA). Briefly, 250 ng of DNA was digested with the appropriate restriction enzyme (*Nsp*I or *Sty*I), PCR amplified, fragmented, and labeled. Microarrays were hybridized, washed, and scanned using a GeneChip 3000 scanner and Affymetrix GeneChip Command Console software. Cell intensity files (.CEL) were generated, saved, and transported to a workstation that contained Affymetrix Genotyping Console (GTC) 4.0 software. To maximize the accuracy of the analysis, we analyzed all samples in a single run, including the 25 control DNAs. SNP CN was calculated based on the hybridization intensity of each SNP probe and was estimated from raw signal data using GTC 4.0. The software compares the tumors with the reference set of normal DNA samples. For this analysis, the protocol of unpaired samples was followed. SNP calls and the analysis of CN were performed using the default parameters of GTC 4.0, and standard deviations for cytogenetic analysis were selected (http://media.affymetrix.com/support/downloads/manuals/gtc_4_1_user_manual.pdf). A 5-state hidden Markov model was applied for smoothing and segmenting CN data. The different states were defined as follows: 0 =  homozygous deletion, 1 =  heterozygous deletion, 2 =  normal diploid, 3 =  single copy gain and 4 =  amplification. As the proportion of genes with CN = 4 was very low (7%) and the tumor recurrence of individual genes with CN = 4 was not greater than 3 tumors, in this study CN 3 and CN4 were considered together in the analysis. The CNCHP output files contained the estimation of CN-altered SNPs in tumors, and the CNAs were defined with the CN segment reporting tool. We selected the segments of DNA with a continuous succession of 50 or more CN-altered SNPs of ≥500 Kb and ≤50% overlap with germ line constitutive CNVs. In addition, the CN alterations were analyzed with the SVS ver. 7.1 software (Golden Helix, Bozeman, Montana, USA) as previously described [23].

### Validation of Genechip Human Mapping 500 K with a Second High-throughput Microarray (Cytoscan HD 2.7)

The CNA profile of 15 samples explored with the 500 K microarray was also examined with an Affymetrix DNA microarray Cytoscan High Density (Affymetrix). This array covers the whole human genome with 2.7 million probes, including 1.9 million non-polymorphic markers. The experiments were performed according to the manufacturer’s standard protocol (Affymetrix). Briefly, 250 ng of tumor DNA was digested with the *Nsp*I restriction enzyme, PCR amplified, fragmented, and labeled. Microarrays were hybridized, washed, and scanned using the GeneChip 3000 7G scanner and Affymetrix GeneChip Command Console software. Cell intensity files (.CEL) were generated, saved, and transported to the Chromosome Analysis Suite software (ChAS) ver. 2.0.1. The CN segments were calculated based on the hybridization intensity of each dual quantile normalized probe with the ChAS. To calculate the log2 ratios, we used a 380-sample data set reference from ChAS, which included 284 HapMap samples and 96 healthy male and female samples. The smoothing and joining methods to create CN segments were set as defaults. CNAs were filtered for losses and gains with a size of ≥500 Kb, with at least 50 altered markers and with ≤50% overlap with polymorphic CNVs.

### Validation of CN of 7 Genes Located in 3q with qPCR

The CNs of 7 genes (*CLDN1*, *PLOD2*, *ECT2*, *NLGN1*, *NAALADL2*, *PLSCR1* and *PLSCR4*) located in 3q were calculated in 31 tumors using 17 samples of lymphocyte DNA as a reference control. Experiments were performed in triplicate on a Rotor-Gene 6000 Corbett detection system (Corbett Life Science, Sydney, Australia), using TaqMan Assays (Applied Biosystems, Foster city, CA) (Table S2) as previously described [23].

### Gene Expression Profiling and Data Analysis

Gene expression profile was explored in 55 CC and 17 cervical epithelium controls using the Human Gene 1.0 ST oligonucleotide microarray (Affymetrix). This array contains 33,297 probe sets that correspond to approximately 21,034 genes of the human gene reference database according to UCSC Genome Browser Assembly Mar. 2009 NCBI 37/hg19 available at http://genome.ucsc.edu/. A total of 300 ng of RNA of each CC or control sample was used, and the procedures for labeling, hybridization, and scanning were performed as previously described [50]. To assess the quality of the experiments, the following parameters were used: the expression of the exogenous polyA controls, the presence of the oligo B2 used to make grid alignments, and the values of the area under the curve above 0.8. Only microarrays with optimal quality controls were analyzed. Furthermore, analysis of some samples was performed in duplicate to evaluate the reproducibility of the experiment, which was higher than 99%. Microarrays were normalized using the robust multichip average algorithm in the Affymetrix expression console. The values of the normalized intensity were referred as units of intensity. The identification of differentially expressed genes in the cancer and control groups was performed with the SAM algorithm (SAM Version 3.0, http://statweb.stanford.edu/~tibs/SAM/) using cutoff values of FC ≥1.5, a general false discovery rate of 0%, and a local false discovery rate of <10% [51]. Unsupervised hierarchical clustering was performed using dChip software (version 1.6, http://www.hsph.harvard.edu/cli/complab/dchip/) following an Euclidean distance metric, average linkage method, and gene ordering by peaking time.

In addition, the expression status (downregulated, upregulated, or without change) for each explored gene (n = 21,034) in each tumor was identified using cutoff values. Upregulated genes were identified when the intensity signal in a given tumor was ≥2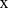 and >
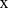
+1*s* of the control group. Downregulated genes were identified when the intensity signal in a given tumor was ≤0.5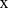 and < 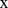-1*s* of the control group.

### Validation of Global Gene Expression with Qrt-PCR

A set of 28 genes was used to validate gene expression in 27 CC and 6 healthy cervical epithelium control samples with qRT-PCR using TaqMan probes. The genes included were *CCNB2, CDC20, CDK1, CDKN2A, CDKN3, CFD, CKS2, CLDN1, ECT2, EDN3, MCM2, MKI67, NLGN1, NUSAP1, PARP1, PCNA, PLOD2, PLSCR1, PLSCR4, PRC1, RFC4, RRM2, SMC4, SYCP2, TOP2A, TYMS, WISP2* and *ZWINT*. *GAPDH* was used as internal control. TaqMan (Applied Biosystems) gene expression assays were used (Table S2). The experiments were run in triplicate on a Rotor-Gene 6000 (Corbett Research) and the procedure was performed as previously described [50].

### Gene Ontology Classification Analysis

The DAVID functional annotation tool (http://david.abcc.ncifcrf.gov/) [52], [53] and IPA (Ingenuity Systems) were used to classify the deregulated genes. Genes were classified using functional annotation clustering considering the gene ontology biological processes. Classification stringency was set at the medium and highest levels.

### Survival Analysis of Cancer Patients

According to FIGO staging, patients with CC received individualized treatment based on the treatment guidelines for CC of the American Cancer Society (see Table 2). After treatment was completed, each patient was clinically evaluated by an experienced oncologist every 3 or 6 months. Clinical data of the follow-up study were obtained from patient medical records. A social worker also made phone calls and home visits to the patients every 6 months during the study. Patients recorded as alive during the study were successfully followed up for an average of 63 months after treatment. Censored and deceased patients were followed up the number of months indicated in Table 2. Cases considered censored were those with patients lost during the follow-up study or who died from causes other than CC. Patients were considered lost when they missed medical appointments for disease control, were not at home for visits, or did not answer or return phone calls. In this cohort, patients recorded as deceased were only those women who died of CC primary tumor as a main cause. The cause of death of all patients who died during the follow up was confirmed with medical records and death certificates. Four patient deaths were excluded from the survival analysis because they refused to receive treatment, leaving 51 patients for the analysis. Of these patients, 1 was considered censored and 16 deaths were registered. The effects of FIGO, total %CN-AG, %CN-AG of 3q, and the gene expression profiles of glycolysis and the APC/C-dependent proteasomal protein catabolic process on survival were investigated through survival analysis, which was performed as previously described [50]. The cumulative overall survival time was calculated using the Kaplan-Meier method and analyzed with the log-rank test. FIGO staging, %CN-AG and the gene expression of the APC-dependent proteasomal protein catabolic process and glycolysis were included as covariates in a Cox proportional hazard model. Ancestry was not included as covariate, since the ancestry proportions [Amerindian (71.1%), European (27.1%) and African (1.8%)] are similar among the patients (data not shown)

### Gene Annotation and Data Analysis

The physical position of genes was mapped according to the UCSC Genome Browser Assembly Mar. 2009 NCBI 37/hg19 available at http://genome.ucsc.edu/. Data analysis was performed using Access 2010 (Microsoft Inc, Seattle, WA, USA). The raw MA data was MIAME (minimum information about a microarray experiment) compliant and have been deposited in NCBI’s Gene Expression Omnibus [54] and are accessible through GEO serie accession number GSE52904 (http://www.ncbi.nlm.nih.gov/geo/query/acc.cgi?acc=GSE52904). All statistical tests were 2 sided, and p-values less than 0.05 were considered statistically significant. Data analysis was performed using Sigma Plot ver. 11 and SPSS ver. 17 softwares.

## Supporting Information

Figure S1 **Validation of GeneChip Human Mapping 500 K (500 K) microarray with high-density microarray HD 2.7.** Shown is the percentage of agreement between the copy number (CN)-altered regions found with the 500 K microarray and those found with the HD2.7 microarray according to the size of the altered segment detected with the 500 K microarray. The black line represents the linear correlation curve, and the Pearson’s coefficient and p value are shown.
(TIF)

Table S1 **Differentially expressed genes in cervical carcinomas compared with normal cervical epithelium.** a. The list of genes was obtained by comparing the cervical carcinomas with the control group. The whole-set analysis used 27 tumors and 17 controls. Comparisons of groups with high and low copy number used 10 tumors in each case and the same control group used for the whole-set analysis. The value of score-(d) corresponds to the whole-set analysis. b. Fold change is calculated by dividing the intensity signals of cervical tumors by that of healthy controls. Empty cells indicate that a gene was not deregulated in the comparison indicated in the column title.
(XLSX)

Table S2 **Expression and copy number assays using TaqMan probes (Applied Biosystems).**
(XLSX)

Table S3 **Status of copy number of the 862 genes deregulated exclusively in high-CN tumors.** a. The values of gene expression (fold change) of all these genes are indicated in Table S1. b. All losses were of 1 copy. Combined genes are those that were deleted in some and amplified in other tumors.
(XLSX)

Table S4 **DAVID functional annotation cluster analysis of common genes differentially expressed in both low- and high-CN tumors as compared with healthy cervical epitheliums.**
(XLSX)

Table S5 **Genes of some biological processes enriched or associated to 3q in the set of genes deregulated exclusively in high-CN tumors.** a. The gene expression value (fold change) of these genes are indicated in Table S1.
(XLSX)
